# Aerobic Exercise Training Leads to MASH Resolution as Defined by the MASH Resolution Index

**DOI:** 10.1007/s10620-025-09361-9

**Published:** 2025-09-03

**Authors:** Theja Channapragada, Sarah Batra, Breianna L. Hummer, Vernon M. Chinchilli, Daniel Huang, Rohit Loomba, Ian R. Schreibman, Jonathan G. Stine

**Affiliations:** 1 Department of Medicine, Penn State Health-Milton S. Hershey Medical Center, Hershey, PA USA; 2 Division of Gastroenterology and Hepatology, Department of Medicine, Penn State Health- Milton S. Hershey Medical Center, 500 University Drive, Hershey, PA 17033 USA; 3 Fatty Liver Program, Penn State Health-Milton S. Hershey Medical Center, Hershey, PA USA; 4 Department of Public Health Sciences, Penn State College of Medicine, Hershey, PA USA; 5 Division of Gastroenterology and Hepatology, Department of Medicine, University of California San Diego, La Jolla, CA USA; 6 Division of Epidemiology, Department of Family Medicine and Public Health, University of California San Diego, La Jolla, CA USA; 7 MASLD Research Center, University of California, Los Angeles, USA; 8 Liver Center, Penn State Health- Milton S. Hershey Medical Center, Hershey, PA USA; 9 Cancer Institute, Penn State Health- Milton S. Hershey Medical Center, Hershey, PA USA

**Keywords:** Metabolic dysfunction-associated steatotic liver disease, Metabolic dysfunction-associated steatohepatitis, Aerobic exercise, MASH Resolution Index

## Abstract

**Purpose:**

Exercise training is recommended for all patients with metabolic dysfunction-associated steatotic liver disease. Whether exercise training improves liver histology independent of body weight loss remains controversial. Given the increasing reliance on non-invasive biomarkers as a surrogate for liver histology, we investigated the relationship between exercise training and improvement in liver histology using the MASH Resolution Index (MASH-RI), a validated composite score of multiple biomarkers, in a post hoc analysis of the NASHFit trial.

**Methods:**

This study randomized adults with biopsy-proven MASH to moderate-intensity aerobic exercise training or standard of care for 20 weeks. Mediterranean-informed dietary counseling was provided to each group. Change in the MASH-RI was measured and compared between the two groups (*n* = 23).

**Results:**

Applying the MASH-RI, those who performed exercise training achieved MASH resolution nearly three times more often (33% vs. 13%, *p* < 0.01) versus those who received standard of care. Exercise training improved individual biomarkers included in the MASH-RI, including ALT, AST, and MRI-PDFF.

**Conclusion:**

Exercise training leads to MASH resolution, as defined by the MASH-RI at greater rates than standard lifestyle counseling. Future research is needed to determine how best to use the MASH-RI as a therapeutic monitoring tool to gauge response to lifestyle intervention.

**Clinical Trial Registration:**

NCT03518294.

## Introduction

Metabolic dysfunction-associated steatotic liver disease (MASLD) remains the leading cause of chronic liver disease in the United States, placing millions of individuals at risk for advanced liver disease and its complications [1, 2]. In May 2024, resmetirom became the first health regulator-approved therapeutic for metabolic dysfunction-associated steatohepatitis (MASH). Despite the availability of a MASH-targeted therapeutic like resmetirom and ongoing clinical development of other novel therapeutics, healthy diet and regular exercise are still the foundation of management in MASLD and MASH [3–6]. It is proven that increased physical activity leads to innumerable benefits, including improvement in hepatic steatosis and hepatic inflammation, independently of body weight loss [7, 8]. Currently, there is no direct scientific evidence, particularly from studies using paired liver biopsies, to confirm that exercise alone can improve liver fibrosis in MASH without accompanying weight loss [6, 9–11].

In recent years, there has been tremendous growth in the development and utilization of non-invasive biomarkers in MASLD and MASH, including those which correlate with improvement in liver histology [12, 13]. Biomarkers of therapeutic response include aspartate aminotransferase (AST), alanine aminotransferase (ALT), vibration controlled transient elastography (VCTE), enhanced liver fibrosis (ELF) test, magnetic resonance imaging proton density fat fraction (MRI-PDFF), and magnetic resonance elastography (MRE), with clinically meaningful thresholds of improvement widely accepted for each [14–17]. Non-invasive biomarkers have significant advantages over liver biopsy, allowing for reliable and repeatable monitoring without procedural risks, minimizing sampling error and variability, and being more cost-effective for widespread clinical use [18]. While non-invasive biomarkers show promise for diagnosing and monitoring therapeutic response clinically, especially in early phase MASH drug studies, they continue to remain investigational under the FDA and require further validation before being considered for regulatory or routine clinical use [19, 20]. While reductions in ALT (≥ 17 U/L) and MRI-PDFF (≥ 30%) are associated with increased odds of histologic improvement in MASH, neither is sufficiently specific to serve as a standalone surrogate, with only modest diagnostic performance [21]. To address this, the MASH Resolution Index (MASH-RI) was recently developed and validated from cohorts of patients with biopsy-proven MASH and liver fibrosis enrolled in MASH drug trials [21]. The MASH-RI combines ALT, AST, and MRI-PDFF to detect MASH resolution without a liver biopsy and offers better accuracy and precision than single biomarkers of response [21].

We recently completed the NASHFit study, which showed a 20-week supervised aerobic exercise intervention reduced plasminogen activator inhibitor‑1 (PAI‑1), a biomarker of thrombotic risk, in adults with biopsy-confirmed NASH. In addition, the study found that adults with biopsy-proven MASH who performed aerobic exercise training for 20 weeks were four times more likely to achieve clinically meaningful improvement in ALT (reduction ≥ 17 IU/L) and three times more likely to achieve clinically meaningful improvement in MRI-PDFF (≥ 30% relative reduction) [9, 22]. Importantly, exercise intervention did not lead to any clinically significant changes in body weight, with an average weight loss of < 3% [9]. Given the emergence of the MASH-RI as a better tool to determine histologic response to therapeutic intervention, we aimed to apply this tool to further investigate the therapeutic benefit of exercise training on non-invasive biomarkers in adults with MASH who underwent an exercise training protocol.

## Patients and Methods

### Study Design and Population

We conducted a post hoc analysis of the previously published 20-week NASHFit study (NCT03518294) which examined the effectiveness of moderate-intensity (40–59% heart rate reserve) aerobic exercise training compared to standard of care [22, 23]. The original study enrolled 28 sedentary adults, with 24 completing the study, between May 2018 and February 2021, who have biopsy-confirmed MASH using NASH Clinical Research Network histologic scoring system [24]. Exclusion criteria involved other chronic liver disease, excessive alcohol consumption, uncontrolled diabetes, or concerns about completing exercise sessions. Adults with MASH were randomly assigned in a 2:1 ratio to receive either the exercise training intervention or standard of care. Patients in the intervention group engaged in guideline-recommended aerobic exercise, completing a total of 150 min of moderate-intensity aerobic exercise weekly, divided into five 30-min sessions [6]. Exercise sessions were directly supervised by a study Exercise Physiologist. Patients in the standard-of-care group continued their regular clinical care as determined by their treating medical provider. All patients received standard lifestyle education and Mediterranean-informed dietary counseling. Digital monitoring and direct supervision of exercise sessions were in place to ensure participant safety and compliance. Clinically significant weight loss was defined as a ≥ 5% reduction in body weight [11]. Outcomes were assessed at baseline and the end of the intervention period; no extended follow-up was conducted beyond the 20 weeks.

All data used in this post hoc analysis were prospectively collected as part of the NASHFit trial. Liver biopsies were obtained within six months prior to enrollment and assessed by a single-blinded pathologist. MRI-PDFF quantified liver fat at baseline and week 20. Fasting labs, including liver enzymes, hemoglobin A1c, glucose, and lipids, were collected at both time points. Body composition was evaluated using dual-energy X-ray absorptiometry (DEXA) and standardized skinfold measurements. NAFLD Fibrosis Score (NFS) and FIB-4 scores were calculated using clinical and laboratory data. Detailed information about research methods completed in this study has been highlighted in previous publications [9, 22, 23]. The study was approved by the Penn State Health Institutional Review Board. All patients provided informed consent prior to being included in the study. All research was conducted in accordance with the Declaration of Helsinki, Good Clinical Practice guidelines, and local regulatory requirements.

This post hoc analysis applied the MASH Resolution Index, which is calculated as follows: 0.520–0.003 × baseline ALT (U/L) − 0.024 × (absolute change in ALT [U/L]) − 0.048 × baseline MRI-PDFF − 2.571 × (percentage change in MRI-PDFF) − 0.039 × baseline AST (U/L) [21]. Change in the MASH Resolution Index was measured and compared between the two groups. Data were available to calculate the MASH Resolution Index in 23 subjects (15 exercise, eight standard of care).

### Endpoints

The primary endpoint of this study was the proportion of patients achieving MASH resolution defined as MASH-RI > − 0.67 after 20 weeks of intervention. Secondary outcomes include > 17 IU/L reduction in ALT, changes in MRI-PDFF, and body composition parameters including BMI, body weight, waist circumference, and body fat percentage.

### Statistical Analysis

Baseline characteristics of participants in the exercise and standard clinical care groups were compared, including anthropometric measures, metabolic parameters, imaging biomarkers, and histologic features. Imaging and serum biomarkers, as well as changes in anthropometric and body composition measures, were evaluated at both baseline and at 20 weeks. Comparisons were made between groups (exercise vs. standard clinical care) and within groups (baseline and at 20 weeks).

The analysis of the primary endpoint was performed with the use of a chi-squared test. Continuous variables were analyzed using paired t tests for within-group comparisons and two-sample t tests for between-group comparisons; other categorical variables were analyzed again by the chi-squared test or Fisher’s exact test where appropriate. Statistical significance was determined by two-sided *p* values of < 0.05. SAS (Cary, NC) Version 9.4 was used for all statistical analysis.

## Results

### Baseline Characteristics

Data were available to calculate the MASH-RI for a total of 23 patients. For participants at study entry, mean body weight was 101 ± 18 kg, and the mean BMI was 33.8 ± 5.0 kg/m^2^. In the study, 57% were female, 39% had diabetes, 83% had hypertension, and 61% had hyperlipidemia. Over 90% were non-Hispanic whites. Moreover, 56% (*n* = 13) had stage F0/F1 fibrosis, 22% had F2 (*n* = 5), and 22% had F3/compensated F4 (*n* = 5). Between the exercise and standard-of-care groups, baseline characteristics such as age, sex, BMI, metabolic disease, liver histology, and non-invasive markers of disease activity were similar (Table 1). All participants had biopsy-confirmed NASH as defined by the NASH Clinical Research Network (NASH CRN) scoring system, with mean NAS scores ≥ 5.

**Table 1:** Baseline comparisons between Exercise and Standard Clinical Care participants

	Exercise (*n* = 15)	Standard Clinical Care (*n* = 8)
Age, yrs	55.2 (10.6)	46.1 (11.0)
Female sex, *n* (%)	10 (67)	3 (38)
BMI, kg/m^2^	33.4 (4.9)	34.6 (5.3)
Body weight, kg	97.2 (13.2)	108.6 (23.0)
Body fat, (%)	43.7 (7.6)	40.5 (11.6)
Diabetes (%)	6 (40)	3 (38)
Hyperlipidemia (%)	9 (60)	5 (63)
Hypertension (%)	14 (93)	5 (63)
Hemoglobin A1c, %	6.4 (1.3)	6.2 (1.1)
Glucose (fasting), mg/dL	131.2 (36.6)	135.6 (54.5)
Liver Specific Testing		
Non-invasive tests		
FIB-4	1.32 (0.48)	1.73 (2.11)
NFS	−1.51 (0.98)	−1.54 (1.88)
Imaging biomarkers		
Liver fat (MRI-PDFF), %	19.7 (5.8)	21.7 (11.6)
NAS	5.1 (1.0)	5.0 (0.5)
Steatosis	2.5 (0.7)	2.4 (0.5)
Lobular inflammation	1.4 (0.6)	1.4 (0.5)
Hepatocyte ballooning	1.2 (0.4)	1.3 (0.5)
Fibrosis stage, *n* (%)		
0/1	9 (60)	4 (50)
2	2 (13)	3 (38)
3	4 (26)	0 (0)
4	0 (0)	1 (12)

*BMI* body mass index, *MRI* magnetic resonance imaging, *NAS* NAFLD Activity Score, *PDFF* proton density fat fraction

^*^Continuous variables reported as mean + / − SD

### MASH Resolution Index

**Fig. 1:**
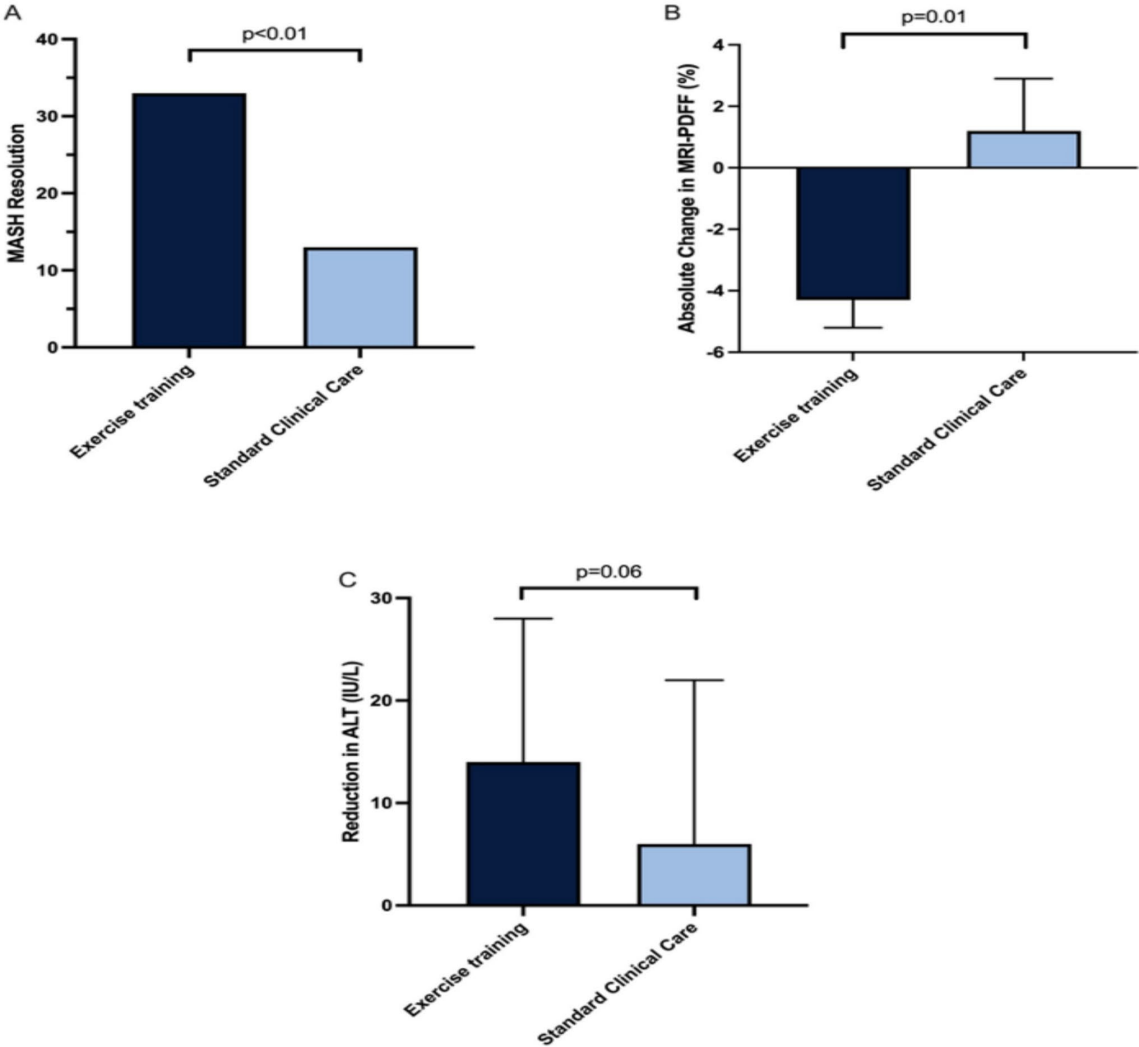
Exercise training leads to greater rates of (**A**) MASH resolution, (**B**) MRI-PDFF improvement, and (**C**) Percentage of participants with ≥ 17 IU/L reduction in ALT comparison to standard of care

More patients in the moderate-intensity aerobic exercise group (33%) experienced MASH resolution compared to standard of care (13%, *p* < 0.01) (Fig. 1). These changes in liver resolution parameters were independent of significant (≥ 5%) body weight loss (Table 3). Each parameter in the MASH-RI also changed independently. The exercise group saw a 24% (−14 ± 14 IU/L) reduction in ALT compared to a 10% (− 6 ± 16 IU/L, *p* = 0.06) reduction for the standard-of-care group (Fig. 1). Fifty three percent of participants in the exercise group achieved a reduction of ALT ≥ 17 IU/L compared to 13% in the standard-of-care group (*p* < 0.01) (Table 2). Exercise training also reduced MRI-PDFF by 4.3% compared to a 1.2% gain for the standard-of-care group (*p* = 0.01) (Fig. 1). Moreover, 36% of the exercise group achieved ≥ 30% relative reduction in MRI-PDFF compared to 13% in the standard-of-care group (*p* = 0.016) (Table 2). In addition, 33% of patients achieved both ≥ 17 IU/L and ≥ 30% relative MRI-PDFF reduction with exercise training compared to 0% who had standard clinical care (Table 2). In relationship to body weight loss, 7% (*n* = 1) of exercise subjects achieved clinically significant body weight loss vs.0% in the standard-of-care group.

**Table 2:** Outcome measures: Biomarkers

	Exercise (*n* = 15)			Control (*n* = 8)			Between group *p* value
	Baseline	Post	Within group *p* value	Baseline	Post	Within group *p* value	
Imaging biomarkers							
MRI-PDFF liver fat, %	19.7 (5.8)	15.4 (4.9)	0.038	21.7 (11.6)	22.9 (13.3)	0.851	0.012
≥ 30% relative reduction in MRI-PDFF, *n* (%)		5 (36)			1 (13)		0.016
Serum Biomarkers							
≥ 17 IU/L reduction in ALT, *n* (%)		8 (53)			1 (13)		< 0.001
Combined imaging + serum biomarkers							
≥ 17 IU/L reduction in ALT + ≥ 30% relative reduction in MRI-PDFF, *n* (%)		5 (33)			0 (0)		< 0.001

*ALT* alanine aminotransferase, *FIB-4* Fibrosis-4 index, *MRI* magnetic resonance imaging, *NFS* NAFLD Fibrosis Score, *PDFF* proton density fat fraction

^*^Reported as mean ± SD

Table 3 summarizes additional anthropometric and body composition changes after exercise training and in comparison to standard of care. In the exercise group, there are modest reductions in BMI, body weight, waist circumference, and body fat compared to the standard-of-care group, with between-group differences in weight (*p* = 0.051) and waist circumference (*p* = 0.069).

**Table 3:** Outcome measures: Anthropometry and Body composition

	Exercise (*n* = 15)			Control (*n* = 8)			
	Baseline	Post	Within group *p* value	Baseline	Post	Within group *p* value	Between group *p* value
BMI, kg/m^2^	33.4 (0.6)	32.8 (0.3)	0.729	34.6 (5.3)	35.0 (5.4)	0.880	0.129
Body weight, kg	97.2 (13.2)	94.9 (12.3)	0.625	108.6 (23.0)	110.1 (23.3)	0.896	0.051
Waist circumference, in	43.7 (3.5)	43.0 (0.6)	0.596	45.8 (4.9)	46.3 (4.8)	0.835	0.069
Body fat, %	43.7 (7.6)	42.3 (8.0)	0.628	40.5 (11.6)	38.4 (11.1)	0.724	0.374

*BMI* body mass index

## Discussion

This post hoc analysis of the NASHFit study applying the MASH-RI showed that adults with MASH who completed aerobic exercise training achieved MASH resolution nearly three times more often than those who received standard of care and without clinically significant body weight loss. This change in composite biomarker provides further confidence in exercise training as a weight-neutral intervention that can improve surrogates of liver histology, the driver of clinical outcomes in patients with MASH [14, 15]. Clinical outcomes in MASH are best captured through liver histology. However, in trials without serial biopsies, especially in lifestyle trials, validated non-invasive biomarkers such as MRI-PDFF and ALT, as well as composite indices like the MASH Resolution Index, serve as practical surrogates that approximate histologic changes. While not perfect substitutes, these surrogates are essential for evaluating responses and minimizing patient risk. This is especially important in the current treatment landscape where histological improvement in MASH activity and/or liver fibrosis is the goal of all therapeutics, including lifestyle-based interventions, and remains required prior to drug approval by the FDA [25]. This is one of the first studies to apply the MASH-RI to exercise training and MASH and helps to support our hypothesis that aerobic exercise training helps to improve multiple biomarkers of liver fibroinflammation and extends our previous studies showing improvement in MRI-measured liver fat and MASH activity [26].

The most widely studied and established benefit of exercise training in patients with MASLD/MASH is an improvement in MRI-measured liver fat [10, 27]. Multiple studies have shown that at least 150 min per week of moderate-intensity aerobic exercise, which includes brisk walking or light cycling, can reduce hepatic steatosis at clinically meaningful thresholds, and this amount of physical activity or its equivalent (e.g., 75 min/week of vigorous intensity aerobic activity) is recommended by multiple leading scientific societies including the AASLD, ACSM, AGA, and EASL [28, 29]. Less evidence exists for exercise training achieving clinically significant improvements in other biomarkers of liver fibroinflammation, including ALT reduction; however, this was shown in the NASHFit study and by others [9, 16].

Although reductions in non-invasive biomarkers such as ALT (≥ 17 U/L) and MRI-measured liver fat have previously been associated with histologic improvement in MASH, their diagnostic performance remains modest, with reported area under the curve (AUC) values below 0.66 [21]. The MASH-RI was developed to overcome these limitations by combining baseline and dynamic changes in ALT, MRI-PDFF, and AST. The MASH-RI was developed and validated from cohorts of patients with biopsy-proven MASH and liver fibrosis enrolled in MASH drug trials [21]. In both derivation and external validation cohorts, the MASH-RI demonstrated superior accuracy for detecting histologic MASH resolution, with AUCs of 0.81 and 0.83, respectively. At the rule-out threshold (≤ − 2.67), the MASH-RI achieved negative predictive values of 92.6% and 100%, while the rule-in threshold (≥ − 0.67) yielded specificities of 89.6% and 53.0%. In the validation cohort, the PPV of the MASH-RI at the rule-in threshold (≥ − 0.67) was 29.2%, which, while modest, outperformed other non-invasive biomarkers such as ALT (PPV 23.7%) and MRI-PDFF (PPV 25.8%). Compared to ALT or MRI-PDFF alone, the MASH-RI provides substantially greater diagnostic precision and consistency. The MASH-RI has recently been compared to other emerging validated tests that help to identify patients with active steatohepatitis and significant fibrosis, such as the FibroScan-aspartate aminotransferase (FAST) score. The FAST score combines liver stiffness measurement (LSM) and controlled attenuation parameter (CAP) from VCTE with AST. In a recent prospective, biopsy-controlled head-to-head study, the MASH-RI significantly outperformed the FAST score in detecting MASH resolution, with a higher AUC (0.83 vs. 0.65, *p* = 0.001) and higher negative predictive value (100% vs. 94%), further supporting its use as a reliable non-invasive tool for monitoring treatment response [30].

Whether exercise training improves liver histology independent of body weight loss remains controversial and of high clinical significance given the inability of most individuals with MASLD/MASH to achieve and sustain body weight loss [31, 32]. Although changes in body weight and waist circumference did not reach statistical significance in our study, between-group differences approached the threshold (*p* = 0.051 and *p* = 0.069, respectively), suggesting a potential directional effect of aerobic exercise on body composition. Importantly, the observed improvements in MASH resolution occurred largely independent of these anthropometric changes. Because the physiologic stress of exercise impacts multiple pathways favorably and simultaneously, offering a therapeutic broadsword mechanistically speaking, it is not surprising that exercise could exert a weight-neutral benefit [33]. Aerobic exercise improves key pathophysiologic mechanisms in MASH by enhancing insulin sensitivity, reducing hepatic fat accumulation, increasing mitochondrial fatty acid oxidation, and modulating pro- and anti-inflammatory cytokines independent of weight loss [33]. Preclinical and clinical data also support exercise-induced attenuation of fibrogenesis through reduced stellate cell activation and oxidative stress [34]. Moreover, improvement in liver fat and MASH resolution precede fibrosis reversal, making our study’s findings of high importance. Based on this, it seems reasonable to conclude that should patients continue to complete guideline-based amounts of aerobic exercise over a longer duration, fibrosis improvement may follow [35, 36].

Additionally, our findings also suggest the need to re-evaluate our approach to supporting individuals with MASH. While weight loss remains a central goal in MASH management, especially with patients having greater access to multidisciplinary weight loss programs, pharmacotherapy, and bariatric surgery, our findings suggest the importance of broadening our focus to include overall clinical improvement. Exercise and dieting can improve MASH independent of significant weight loss, and emphasizing these benefits may help support more sustainable lifestyle changes in clinical practice, thereby enhancing patient motivation and engagement in long-term care. We have evidence to suggest that the MASH-RI can be used as a therapeutic monitoring tool alongside other non-invasive biomarkers to determine response to lifestyle intervention and one that clinicians can rely on when convincing patients about the importance of achieving their weekly dietary and physical activity goals [25, 37].

Potential limitations of this study include modest sample size, primarily non-Hispanic White population, and relatively short intervention and follow-up duration, as well as the exploratory nature of a post hoc analysis, which was not included in the original study protocol. Limitations inherent to the MASH-RI include a lack of data to inform on its ability to predict clinical outcomes, such as progression to cirrhosis or HCC, and no post-intervention liver biopsies being performed. Strengths, however, are numerous and include excellent exercise adherence (> 80%) and improvement across multiple important clinical endpoints. Additionally, the application of the MASH-RI, which was derived based on pharmacologic studies utilizing an anti-steatotic medication, to a lifestyle intervention trial, further increases its utilization as a combination biomarker.

## Conclusion

This post hoc analysis of the NASHFit study demonstrated that aerobic exercise training achieved MASH resolution three times more often compared to standard clinical care. The application of the MASH-RI provides a novel, non-invasive method to monitor response to lifestyle interventions. Future studies are needed to determine how repeated, longitudinal change in the MASH-RI is related to improvement in both liver fibrosis and clinical outcomes.

## Data Availability

Data from the NASHFit study are available publicly and can be found at https://www.icpsr.umich.edu/web/pages/.

## Abbreviations

ALT: Alanine Aminotransaminase
AST: Aspartate Aminotransferase
BMI: Body mass index
ELF: Enhanced Liver Fibrosis
FNI: Fibrotic NASH Index
HCC: Hepatocellular carcinoma
MASLD: Metabolic dysfunction-associated steatotic liver disease
MASH: Metabolic dysfunction-associated steatohepatitis
MASH-RI: MASH Resolution Index
MRI: Magnetic resonance imaging
PDFF: Proton density fat fraction
VCTE: Vibration Controlled Transient Elastography

## Declarations

### Permission to reproduce material from another source

No material from another source was reproduced.
